# Micro to macro scale analysis of the intact human renal arterial tree with Synchrotron Tomography

**DOI:** 10.1101/2023.03.28.534566

**Published:** 2023-03-29

**Authors:** Shahrokh Rahmani, Daniyal J. Jafree, Peter D. Lee, Paul Tafforeau, Joseph Jacob, Alexandre Bellier, Maximilian Ackermann, Danny D. Jonigk, Rebecca J. Shipley, David A. Long, Claire L. Walsh

**Affiliations:** 1 Department of Mechanical Engineering, University College London, London, UK, WC1E 6BT; 2 Developmental Biology and Cancer Research & Teaching Department, UCL Great Ormond Street Institute of Child Health, University College London, London, UK, WC1N 1EH; 3 UCL MB/PhD Programme, Faculty of Medical Science, University College London, London, UK, WC1E 6BT; 4 European Synchrotron Radiation Facility, Grenoble, France, 38043; 5 Satsuma Lab, Centre for Medical Image Computing, UCL, London, UK; 6 Lungs for Living Research Centre, UCL, London, UK; 7 Department of Anatomy (LADAF), Grenoble Alpes University, Grenoble, France, 38058; 8 Institute of Anatomy, University Medical Center of the Johannes Gutenberg University Mainz, Mainz, Germany; 9 Institute of Pathology and Department of Molecular Pathology, Helios University Clinic Wuppertal, University of Witten-Herdecke, Wuppertal, Germany; 10 Institute of Pathology, RWTH Aachen Medical University, Aachen, Germany; 11 German Center for Lung Research (DZL), Biomedical Research in Endstage and Obstructive Lung Disease Hannover (BREATH), Hannover, Germany

## Abstract

**Background:**

The kidney vasculature is exquisitely structured to orchestrate renal function. Structural profiling of the vasculature in intact rodent kidneys, has provided insights into renal haemodynamics and oxygenation, but has never been extended to the human kidney beyond a few vascular generations. We hypothesised that synchrotron-based imaging of a human kidney would enable assessment of vasculature across the whole organ.

**Methods:**

An intact kidney from a 63-year-old male was scanned using hierarchical phase-contrast tomography (HiP-CT), followed by semi-automated vessel segmentation and quantitative analysis. These data were compared to published micro-CT data of whole rat kidney.

**Results:**

The intact human kidney vascular network was imaged with HiP-CT at 25 μm voxels, representing a 20-fold increase in resolution compared to clinical CT scanners. Our comparative quantitative analysis revealed the number of vessel generations, vascular asymmetry and a structural organisation optimised for minimal resistance to flow, are conserved between species, whereas the normalised radii are not. We further demonstrate regional heterogeneity in vessel geometry between renal cortex, medulla, and hilum, showing how the distance between vessels provides a structural basis for renal oxygenation and hypoxia.

**Conclusions:**

Through the application of HiP-CT, we have provided the first quantification of the human renal arterial network, with a resolution comparable to that of light microscopy yet at a scale several orders of magnitude larger than that of a renal punch biopsy. Our findings bridge anatomical scales, profiling blood vessels across the intact human kidney, with implications for renal physiology, biophysical modelling, and tissue engineering.

## INTRODUCTION

The kidney receives up to 20% of cardiac output, carried into the organ by arteries branching off the abdominal aorta and entering the renal hilum. Once within the kidney, the renal arteries divide hierarchically, into interlobar and interlobular vessels enroute to the renal cortex and, within the cortex, as arcuate arteries and efferent arterioles. This network perfuses specialised glomerular capillaries for plasma ultrafiltration, before peritubular capillaries and vasa recta facilitate dynamic solute exchange. Thereafter, venous return follows the arterial supply out of the organ.^1^

Structural and molecular changes to the renal vasculature accompanied by alterations in tissue oxygenation occur in diabetes, hypertension, transplant rejection and chronic kidney disease in both animal models and patients.^2^ Studying renal vascular structure and heterogeneities thus has implications for understanding the basis of renal function in health and disease. Advances in imaging modalities such as micro-computed tomography (μCT),^3–5^ magnetic resonance imaging (MRI)^6^, lightsheet microscopy (LSM)^8,9,^ and photoacoustic imaging^10^ have generated detailed analyses of blood vessels in intact mammalian kidneys, particularly rodents.^5^ Although LSM has been used to quantify cortical vessel diameters in humans,^11^ it has not captured the intact renal vascular network of the human kidney without tissue subsampling.

Here, we map the intact arterial network of an entire human kidney using Hierarchical Phase-contrast Tomography (HiP-CT).^12^ Previously, we quantified glomerular morphology across cubic centimetres of intact human kidney with HiP-CT^12^ and now we extend HiP-CT analysis to the topology of the intact human renal arterial network. We compare topological metrics between rodent and human kidneys and identify spatial heterogeneities that may contribute to oxygenation gradients within the intact human organ.

## METHODS

### Sample preparation

An intact human right kidney was obtained from a 63-year-old male (cause of death: pancreatic cancer) who consented to body donation to the Laboratoire d’Anatomie des Alpes Françaises, (Grenoble, France) before death. Post-mortem study was conducted according to Quality Appraisal for Cadaveric Studies scale recommendations.^13^ The body was embalmed by injecting 4500 mL of 1.15% formalin in lanolin followed by 1.44% formalin into the right carotid artery, before storage at 3.6 °C. During evisceration of the kidney, vessels were exposed, and surrounding fat and connective tissue removed. The kidney was post-fixed in 4% neutral-buffered formaldehyde at room temperature for one week. The kidney was then dehydrated through an ethanol gradient over 9 days to a final equilibrium of 70%.^14^ The volume of each dehydration solution was four-fold greater than the volume of the organ and during dehydration, the solution was degassed using a diaphragm vacuum pump (Vacuubrand, MV2, 1.9m3/h) to remove excess dissolved gas. The dehydrated kidney was transferred to a polyethylene terephthalate jar where it was physically stabilised using a crushed agar-agar ethanol mixture, and then imaged.^12,14^

### Scanning, image acquisition and reconstruction

Imaging was performed on the BM05 beamline at the ESRF following the HiP-CT protocol.^12,14^ Initially the whole kidney was imaged at 25 μm/voxel (edge length). Volumes of interest within the same kidney were also imaged at 6.5 and 2.6 μm/voxel.^12^ Tomographic reconstruction was performed^12,14^ using PyHST2 software^15^. Briefly, a filtered back-projection algorithm with single-distance phase retrieval coupled to an unsharp mask filter was applied to the collected radiographs. The reconstructed volumes at all three scales were binned (average binning) to 50, 13, and 5.2 μm^3^/voxel, respectively, to reduce computational load for subsequent image segmentation and quantification. (Scanning parameters can be found in Supplementary Table S1)

### Image filtering, enhancement, and segmentation

Prior to semi-automated segmentation, images were filtered to enhance blood vessel wall contrast. A 3D median filter (iterative and 26-adjacent analysis was used to reduce image noise; image normalisation was performed using background detection correction (Amira v2021.1; type: B-spline, voxel dimensions: 91, 130, 227). Semi-automated segmentation of the arterial networks was performed in Amira v2021.1 using a region growing tool. In this method, the user selects seed locations and an intensity threshold, then any pixels within the connected neighbourhood of the seed point and within the threshold are added to the region, and this continues in an iterative fashion expanding the region. The annotator continues to select seed points and thresholds until the interior of all vessels are filled. This method was applied to segment the whole renal arterial network from the intact human kidney from the imaging data at 50 μm^3^/voxel, and portions of the same network in the 13 and 5.2 μm^3^/voxel datasets. The segmentations were proof read by a second independent annotator and segmentation between the initial and the second annotator were iterated until no further changes were found. Arteries and veins, were distinguished due to the thicker walls of arteries, and ultimate connectivity to the renal artery.

### Visualization and skeletonization

To quantify the human kidney blood vasculature, the segmented 3D vascular network at 50 μm^3^/voxel was skeletonized using the centreline tree algorithm in Amira (tube parameters: slope = 6 and zeroval = 16). The resulting spatial graph describes the vascular network in terms of ‘points’, ‘segments’, and ‘vessels’ with definitions for each shown in Fig.S1A. A vessel was defined as the connector between a start and end point, which corresponds to either a branching point leading into another vessel branch or a terminal end where no further branches were detectable. Segments are assumed to be cylinders with a circular cross section joining points that discretise the vessel length.

### Morphological analysis

**Figure 1.**
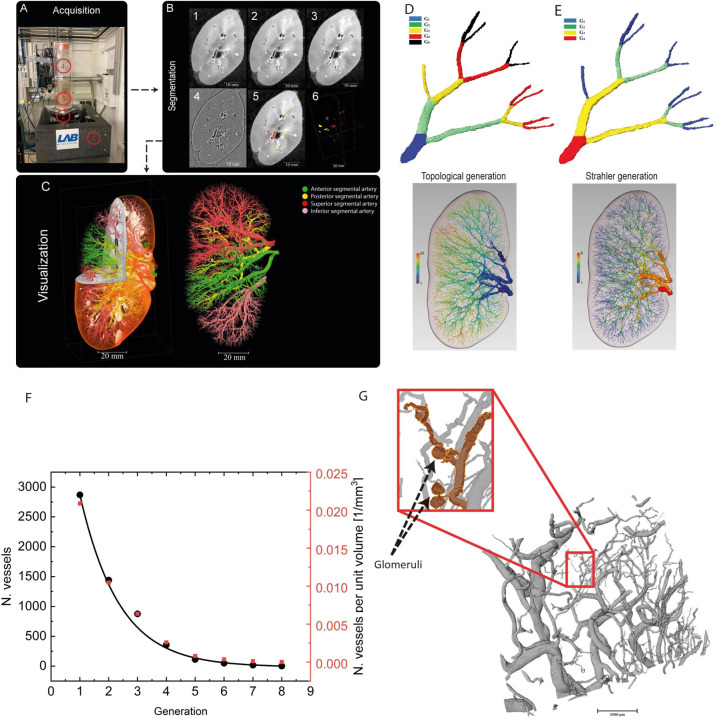
Processing pipeline for HiP-CT imaging and quantitative analysis of the human kidney vasculature. **(A)** Setup for imaging acquisition using HiP-CT at BM05; 1) tomographic stage, 2) platform, 3) sample, 4) reference sample **(B) Image processing pipeline; 1)** a 2D reconstructed image at 25 μm^3^/voxel resolution; 2) binning the image by 2, 3) applying 3D median filter to increase signal to noise ratio, 4) Image normalization using background detection correction, 5) Segmentation and thresholding, 6) Labelling the four main arterial branches **(C)** 3D rendering of the segmented vascular network of a human kidney. Each of the main four branching of the renal artery entering the kidney are colour-coded **(D)** Graphical representation of topological generations within the human kidney vasculature, where each generation (G) is defined by its distance from the main branches of the renal artery (G_1_) **(E)** Graphical representations of Strahler generations within the human kidney vasculature, where each generation (G) is defined by its distance from the smallest calibre vessels detected at this resolution; the interlobular arteries (G_1_) **(F)** Number of vessels and number of vessels per unit volume of the whole kidney by Strahler generation. **(G)** Segmentation of high-resolution (5.2 μm^3^/voxel ) HiP-CT data within a randomly selected cortical region within the human kidney. Glomeruli are visible at the distal tips of the smallest vessels detected at this resolution.

To assess vessel generations, two methods were applied, i) a ‘topological’ approach, starting from the renal artery as generation one, with each branching point increasing the generation number by one (Fig.1D); ii) the Strahler ordering system, where the terminal ends of the network are assigned as the first order apart from a single manually selected point which is designated as the root point (the renal artery in this case), iterating through the network from these end points, at every branch points, when two vessels with the same order intersect, the resulting vessel has an order one greater. Alternatively, if two vessels with different orders intersect, the higher generation of the two is given to the resulting vessel (Fig.1E). Morphological metrics of the network were calculated from the spatial graph as follows (see also Figure S1A):

1. Branching angle: the angle between the two daughter vessels from a common parent vessel, where the vessel vectors are considered to be the Euclidean path between the common branching point of the parent and the two daughters and the end points for each daughter vessel
2. Tortuosity: the Euclidean distance between the start and end points of a vessel divided by the sum of all lengths of segments that make up that vessel,
3. Radius: calculated for each vessel as the mean of all segment radii, or in the case of vessel partial collapse, the maximum segment radius or in the case of complete vessel collapse, an equivalent radius for the perimeter of vessel cross-section in the binary image.
4. Length: the sum of all distances between points in a single vessel
5. Branching ratio: the number of vessels of a given generation/order divided by the number vessels of the order or generation above.
6. Adherence to Murray’s Law: this states that the cube of the parent vessel diameter should be equal to the sum of the cubed daughter vessel diameters, and was assessed by analysing the measurements of vessel diameters and branching points. Murray’s Law suggests that the blood flow in a network is distributed in a manner that minimizes resistance to flow when the diameters of the parent and daughter vessels are in a certain ratio.
7. Inter-vessel distance, used to infer tissue perfusion and oxygenation across the human kidney due to its correlation with diffusion distance, was calculated as the distance of every tissue voxel from its nearest vessel voxel *via* a 3D distance transform applied to the binary vessel image. All codes for the calculation of the above metrics are provided in Supplementary Data.

These metrics can provide insights into the mechanisms underlying the formation and maintenance of the vascular network, as well as the functional implications of deviations from Murray’s Law. Additionally, the above metrics in our human kidney were compared to those of the rat kidney taken from Nordsletten et al.^5^ scanned at 20 μm^3^/voxel using a micro CT scanner, where a radiopaque silicone polymer was perfused to enhance contrast.^5^

### Kidney Compartment Segmentation

Segmentation of the compartments within the human kidney, including cortex, medulla and hilum, was performed in Dragonfly (version: 2021.3) using the segmentation wizard to train a 2D convolutional neural network (CNN). The final hyperparameters of the CNN are given in Table S2. Manual correction of the CNN output was performed in Amira (v2021.1), to provide the final compartment segmentation. These segmentations were used to compare the inter-vessel distance variation between the cortex, medulla, and hilum.

### Statistical analysis

Statistical comparisons of length and radius between human and rat kidneys^5^ was performed in GraphPad Prism (version: 2021), and all graphs and plots were created with Origin 2021b. For statistical comparison of normalised vessel radius and length , a *p* value of less than 0.05 was considered statistically significant. Log of radius and length were plotted against Strahler generation for each of the human and rat datasets, enabling a linear least squares regression. A sum of squares *F* test was performed with the null hypothesis that a single set of global parameters for slope and intercept would fit vessel radius or vessel length for both the rat and human cases. In the case of vessel radius *p* < 0.0001; F (DFn, DFd) = 700.6 (2, 12). In the case of vessel length *p =* 0.4213; F (DFn, DFd) = 0.9299 (2, 12).

## RESULTS

### HiP-CT maps the entire blood vascular network in the intact human kidney

Using HiP-CT^12,14^ (Fig. 1A), we imaged the intact kidney of a 63-year-old male organ donor at 25 μm^3^/voxel . After 3D reconstruction and pre-processing, we segmented all visible renal arteries/arterioles thus extracting the entire arterial network of the organ (Fig. 1B). By generating 3D renderings of the arterial network, we identified a segmental pattern of anterior, posterior, superior and inferior territories supplying the renal parenchyma (Supplementary Video 1). Each vascular territory (shown in different colours in Fig. 1C) had a corresponding renal arterial branch originating from the hilum which bifurcated before hierarchical branching towards the cortical parenchyma.

The vascular dataset consisted of a spatial graph containing 5730 end or branching points, 303,595 points, and 5,718 vessels.

Generational analysis of the network was applied to classify the vessels into established biological hierarchies. We resolved 24 topological generations, or eight Strahler generations, with an exponentially increasing branching ratio down to the eight generations (Fig. 1F). We mapped each generation to established anatomical groups: Strahler generations 7–8 (*n =* 17 vessels; mean radius = 1249 ± 797 μm) mapped to the branches of the renal artery entering the kidney hilum. Generations 5–6 comprise interlobar arteries (*n = 160* vessels; mean radius = 320 ± 131 μm), and generations 2–4 arcuate arteries (*n =* 2672 vessels; mean radius = 90 ± 52 μm). Interlobular arteries fall within generations 1–3 (*n =* 5185 vessels; mean radius = 59 ± 27μm).

The human kidney appeared to have a, lower number of generations than previously found in rat^5^ (8 in human as compared to 11 in rat). To understand this discrepancy, we examined selected areas of the human kidney at higher resolution. We segmented arterioles from selected regions of interest within the same human kidney scanned at 2.6 μm^3^/voxel. From these high-resolution images, we were able to segment a further three generations, corresponding to efferent and afferent arterioles as evidenced by the presence of glomeruli terminal ends of the arteriolar network (Fig.1G). Thus, we are able to verify that HiP-CT of the whole human kidney can resolve the renal arterial network down to the level of interlobular arteries, and use HiP-CTs higher local resolution, to verify that the number of branching generations in the human kidney matches that of the rat.^5^

### Analysis of vascular network metrics in the human kidney reveals concordance with a rodent model organism

Vascular network metrics provide a means for quantitative comparison between individuals, species or pathologies^16,17^ and are increasingly used for the simulation of oxygenation, drug delivery, or generating realistic vascular networks for in silico medical trials.^18,19^ These applications require metrics including branching angle, radius, tortuosity and length as inputs, and often assume adherence to e.g. Murray’s Law. To our knowledge, no such metrics exists for the human kidney, and thus we report them here (Fig. S1B–E, Table 1).

**Figure 2.**
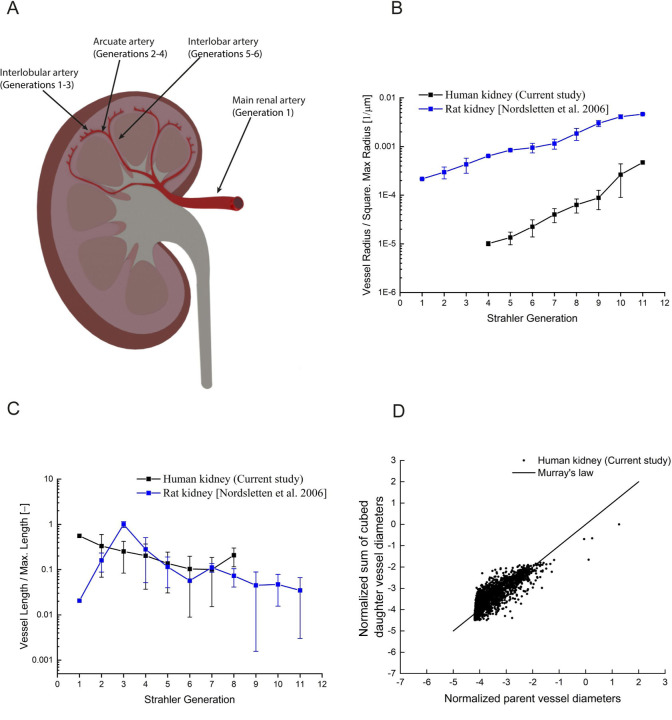
Quantitative comparison of vascular branching metrics in human as compared to rat kidney. **(A)** Plot of normalised vessel radius *vs.* Strahler generation for the human kidney vasculature (black) as compared to rat (blue). Normalisation was performed by dividing the vessel radii by the maximum radius squared in the network. Compared to rats, the normalised vessel radius was much lower in humans at each generation (p < 0.0001) **(B)** Plot of normalised vessel length vs. Strahler generation for the human kidney vasculature (black) as compared to rat (blue). Normalisation was performed by dividing the vessel lengths by maximum vessel length in the network. Normalised vessel lengths were similar between human and rat kidney at each generation (p = 0.4213) **(C)** Assessment of the adherence of the human kidney vasculature to Murray’s law. Normalized parent vessel diameter cubed is plotted against the sum of the cubed normalized daughter vessels; both axes were normalized by the maximum cubed parent vessel diameter. Using linear regression, the deviation between the line of best fit was 4.5%. In **A** and **B**, each data point represents the mean value detected at each generation and error bars represent SEM.

Previously, computational models of mammalian renal blood flow have been derived from quantitative analysis of micro-CT images of the intact rat kidney vasculature^20–22^ until now, it is not been possible to assess how these analyses compare to the human organ. We therefore compared our human kidney HiP-CT data with those derived from micro-CT data of rat,^5^ relating normalised vessel metrics from each species at corresponding generations of the renal arterial network. The increasing trend in vessel radius with generation from interlobular arteries to major renal artery branches was similar between human and rat kidney (Fig. 2B), albeit there was a significantly lower normalised vessel radius at each generation in humans as compared to rat (*p <* 0.0001).^5^ Conversely, normalised vessel lengths were similar between human and rat kidney at each generation (Fig. 2C, *p* = 0.4213).

Branching ratio provides an indication of network symmetry, with a perfectly symmetrical network having a branching ratio of 2. It is hypothesised that the symmetry or asymmetry of a blood vascular network is indicative of constraints imparted upon its architecture by metabolic demands.^23,24^

We found that the branching ratio of the human arterial network was 2.41 whilst the rat kidney is reported as 2.85, ^5^ indicating that both human and rat kidney possess slight branching asymmetry as do many other mammalian networks e.g. the rat lung (3.31) and human torso. ^5,24^

Murray’s law which prescribes the vessel diameter ratio for parent and child vessels that minimises resistance to blood flow, is an often used constraint in branching structure growth simulations.^25^ We demonstrate agreement with Murray’s Law (Fig.2D, *r =* 0.95, R^2^ = 0.758) for the human arterial renal network, as has previously been demonstrated for the rat.^5^ In summary, branching metrics and trends are conserved between the rodent and human kidney. However, certain metrics such as vessel radius vary; an important consideration when extrapolating or translating vascular analyses from rodent to human.

### Regional heterogeneity in vascular branching metrics provides a basis for local hypoxia within the kidney microenvironment

Regional heterogeneity within the kidney creates local microenvironments that enable specialised renal functions. The renal medulla possesses low oxygen tension, generating hypoxia that is inherent to the medulla’s urinary concentration mechanisms. A longstanding hypothesis, supported by MRI studies,^26^ is that vascular rarefaction in acute and chronic kidney diseases results in hypoxia within the renal cortex, stimulating neighbouring cells into a pro-fibrotic phenotype and manifesting in loss of organ function.^2^ Mapping the regional anatomical layout of the vasculature is fundamental to understand local microenvironments, including the generation of physiological, or susceptibility to pathological hypoxia.

**Figure 3.**
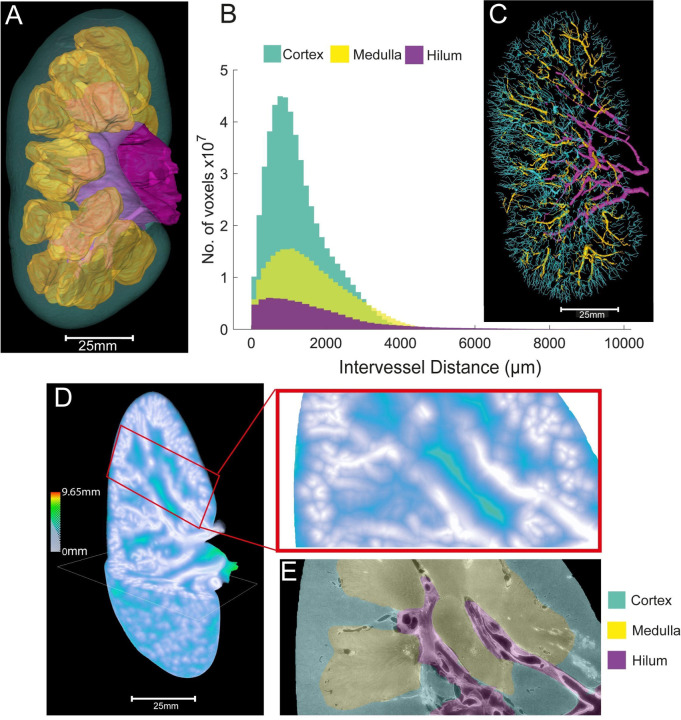
Analysis of zonal heterogeneity in vascular branching metrics within the human kidney. **(A)** 3D reconstruction of semi-automatically generated masks of different zones within the human kidney including cortex (blue), medulla (yellow) and hilum (purple) **(B)** Inter-vessel distances are plotted against the total number of vessel voxels for each kidney zone **(C)** Segmented vasculature from each of the three zones, colour-coded according to zone as in **A (D)** Visual heatmap of inter-vessel distance for the entire human, where red represents the largest inter-vessel distance (> 9.65 mm) and grey (0 mm) the smallest. A zoomed 2D region of interest within cortex and medulla is shown in the red boxed region. **(E)** 2D reconstruction of the HiP-CT image associated with the zoomed region of interest above in **D.**

To assess regional vascular heterogeneity in the human kidney, HiP-CT scans were semi-automatically segmented into hilar, medullary and cortical zones (Fig. 3A). The volume of each zone in addition to the number of vessels, length, radius and volume of segmented vessels within each zone were quantified (Table 2). Despite the majority of the volume of the human kidney being occupied by the cortex (64.7%) as compared with the medulla (27.3%) or hilum (8.0%), the cortex had the lowest vessel volume (9.5% *vs.* 14.3% *vs.* 42.8%). (NB total percentage does not sum to 100%, as vessels that straddle two regions are excluded). As a proxy for renal tissue oxygenation, we quantified (Fig. 3B–C) and mapped (Fig. 3D–E) the inter-vessel distance, reflecting the extravascular distance across which oxygen and solutes diffuse, compartmentalised by hilum, medulla, and cortex. Mean inter-vessel distances for medulla, cortex and hilum (Table. 2) show that the cortex has the lowest inter-vessel distance (1.3 × 103 ± 824 μm) followed by the hilum (1.5 × 103 ± 1400 μm) and medulla (1.6 × 10^3^ ± 1000 μm), thus following anticipated distributions. Notably, there were large portions within the medulla where inter-vessel distance was > 4.5 mm (Fig. 3D), in line with the hypoxic character of the medulla. Whilst the cortex has the lowest inter-vessel distance, small areas with high inter-vessel distance > 4.5mm were found, predominantly towards the renal capsule, which we attribute to the previously discussed resolution limit of these data (see Fig. 1G).

## DISCUSSION

Owing to the limited volume of tissue imageable using modalities such as micro-CT and LSM, and comparatively low resolution of routine clinical imaging, it has been impossible, until now, to capture the entire vascular network of the intact human kidney. We have overcome these limitations using HiP-CT, enabling 3D imaging and segmentation of an entire human kidney arterial network at twenty-fold greater resolution than conventional clinical CT scanners (200 μm voxels), instead comparable to that of light microscopy (1–8 μm pixels) yet at a scale several orders of magnitude larger than that of a renal punch biopsy. The balance between imaging volume and resolution afforded by HiP-CT thus bridges the scale between local cellular structures and global tissue changes, providing quantitative vascular metrics from an intact human organ for the first time. The metric we report here are the first of their kind and thus represent a baseline for the morphology of the human renal arterial network. As further studies are performed, our data will provide a benchmark for natural the variation in human anatomy and also for pathological variations from e.g. diabetes or renal cancer.

Using HiP-CT, we show that the human kidney’s arterial network is exquisitely organised across the organ, with branching metrics optimally arranged to minimise resistance to flow akin to rodent species, whilst also possessing regional heterogeneity likely contributing to physiological gradients in local oxygen tension and susceptibility to hypoxia. Beyond biophysical modelling and providing a platform to study renal pathologies, the dataset generated has immediate practical applications, such as providing inputs for bioprinting of artificial kidneys^27^ or planning tumour resection whilst preserving renal function.^28^

Limitations of this work include the low throughput of the pipeline, the resolution limit of the organ-wide scan, and the limited access to the technique, currently. Emerging solutions to these limitations are provided by, i) machine learning methods for automated segmentation of vessels from imaging data,^29,30^ ii) improvements of the ESRF beamline which are already extending the resolution limit for whole organs down to 8 μm; iii) the release of the data through the Human Organ Atlas portal (https://human-organ-atlas.esrf.eu/). Ultimately, we envisage that mapping microstructural detail will become possible at the scale of the whole kidney, providing a means to link cellular events with organ physiology and pathology.

## Supplementary Material

Supplement 1 **Supplementary Figure 1: (A)** Schematic picture showing blood vessel metrics measured within the human kidney. Vessel: is defined as being between a start and end point; which correspond to either a branching point leading into another vessel branch or a terminal end where no further branches were detectable. Segments: are joining points that discretise the vessel length. Branching angle (α): is defined as the angle between the two vessels, which is measured from the branching point to the ending points of each vessel. Tortuosity (𝞽): is defined as the shortest distance between start and end points of a vessel dividing by the vessel length **(B)** Branching angles distribution (Number of Branching points = 2836, mean = 61.6 ± 29.6°) **(C)** Tortuosity (Number of vessels = 5718, mean = 1.35 ± 0.27) **(D)** Vessel radius against Strahler generation **(E)** Vessel radius over vessel length against Strahler generation

Supplement 2 **Supplementary Video 1:** Animated 3D renderings of the arterial network, showing segmental pattern of anterior, posterior, superior and inferior territories supplying the renal parenchyma

## Figures and Tables

**Table 1.** Human kidney vascular branching metrics by Strahler generation

Generation order	Number of vessels	Radius [μm]	Length [μm]
1	2869	45.7 ± 4.6	4775.9 ± 2116.1
2	1440	61.0 ± 17.7	2312 ± 1961.9
3	876	101.5 ± 38.9	2377.7 ± 2172.9
4	356	181.5 ± 58.5	3159.2 ± 2456.1
5	111	285.5 ± 91.4	4655.6 ± 3808.3
6	49	397.8 ± 170.4	5802.7 ± 3882.1
7	16	1195.6 ± 789.5	7638.8 ± 6072.1
8	1	2124	12792.8

**Table 2.** Human kidney vascular branching metrics by zone

	Cortex	Medulla	Hilum	Organ
Volume of tissue / μm3	8.85 × 10^13^ 64.7%	3.37 × 10^13^ 27.3%	1.09 × 10^13^ 8.0%	1.37 × 10^14^ 100.0%
Number of vessels (Vessels that belong to and cross over two regions were not included in this analysis.)	2758 48%	881 15.4%	115 2.0%	5719 100.0%
Total vessel length / μm ± STD	9.40 × 10^8^	1.95 × 10^6^	6.28 × 10^5^	2.12 × 10^7^
Mean vessel radius / μm ± STD	51.5 ± 22	115.0 ± 61	350.0 ± 184	76.0 ± 68
Mean inter-vessel distances / μm ± STD	1.3 × 10^3^ ± 824	1.6 × 10^3^ ± 1000	1.5 × 10^3^ ± 1400	1.3 × 10^3^ ± 952

## SIGNIFICANCE STATEMENT

High-resolution, three-dimensional, renal vasculature models are currently highly reliant on data obtained from rodent kidneys. Obtaining this information in a human kidney is difficult, given its size and scale. Here, we overcome this challenge through synchrotron-based imaging to profile the vasculature of an intact human kidney. Organ-wide vascular network metrics are shown to be largely conserved between human and rat kidneys. Regional and spatial heterogeneities between cortical, medullary, and hilar vascular architecture are revealed, highlighting a structural basis for renal oxygen gradients in humans. This is, to our knowledge, the first time the vasculature of a human kidney has been mapped in its entirety, with implications for understanding how the hierarchy of individual blood vessel segments collectively scales to renal function.
