# Supplementary figures and images for: Mapping the blood vasculature in an intact human kidney using hierarchical phase-contrast tomography
Source: bioRxiv. 2024 Jul 5:2023.03.28.534566. Originally published 2023 Mar 29. Preprint. [Version 2] doi: 10.1101/2023.03.28.534566 (PMC10081185; doi:10.1101/2023.03.28.534566)

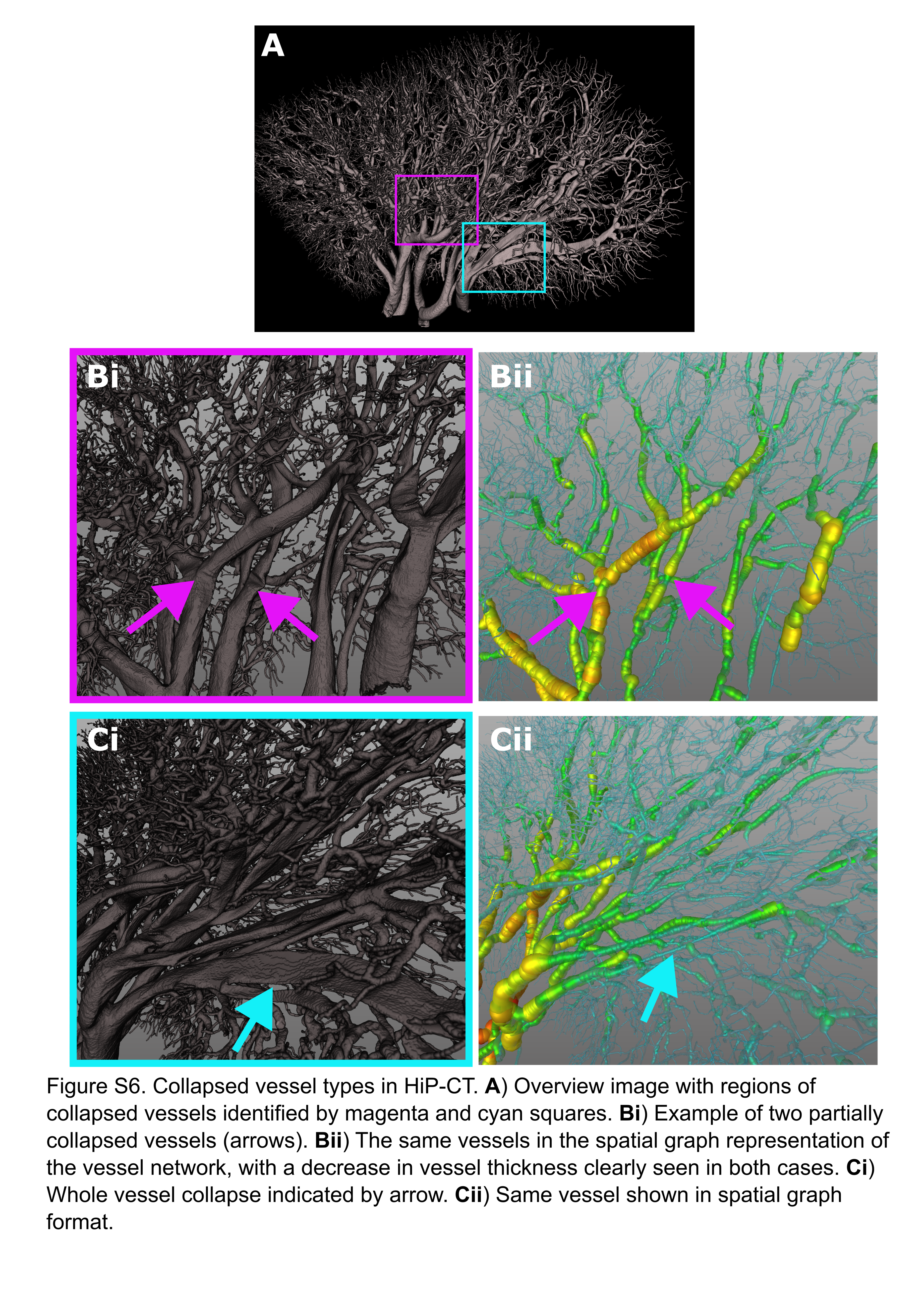

Supplement: Supplement 3 [file media-3.zip › Final_ Supplemenrary_figure_S6.tif]

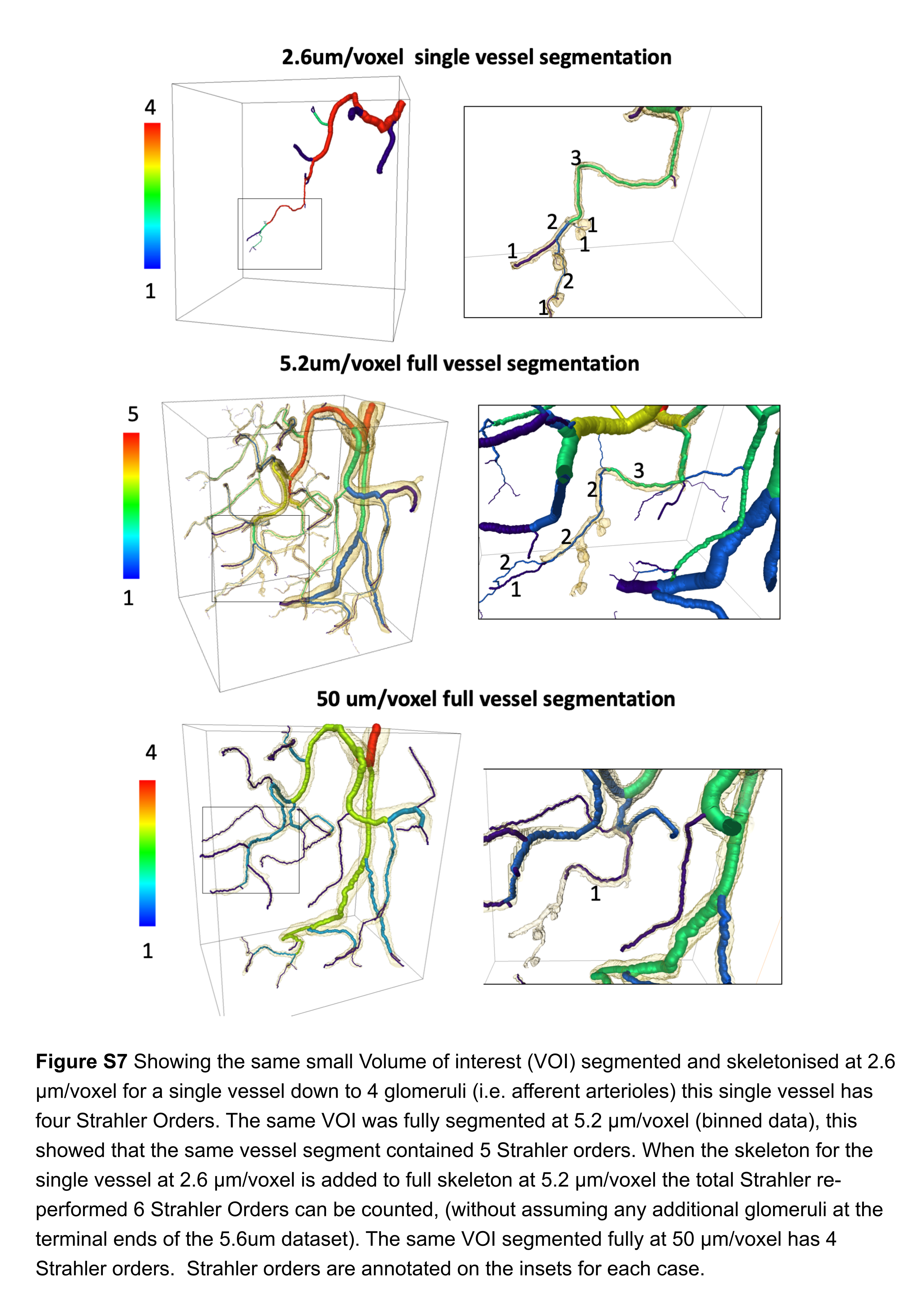

Supplement: Supplement 3 [file media-3.zip › Final_Supplemenrary_figure_S7.tif]

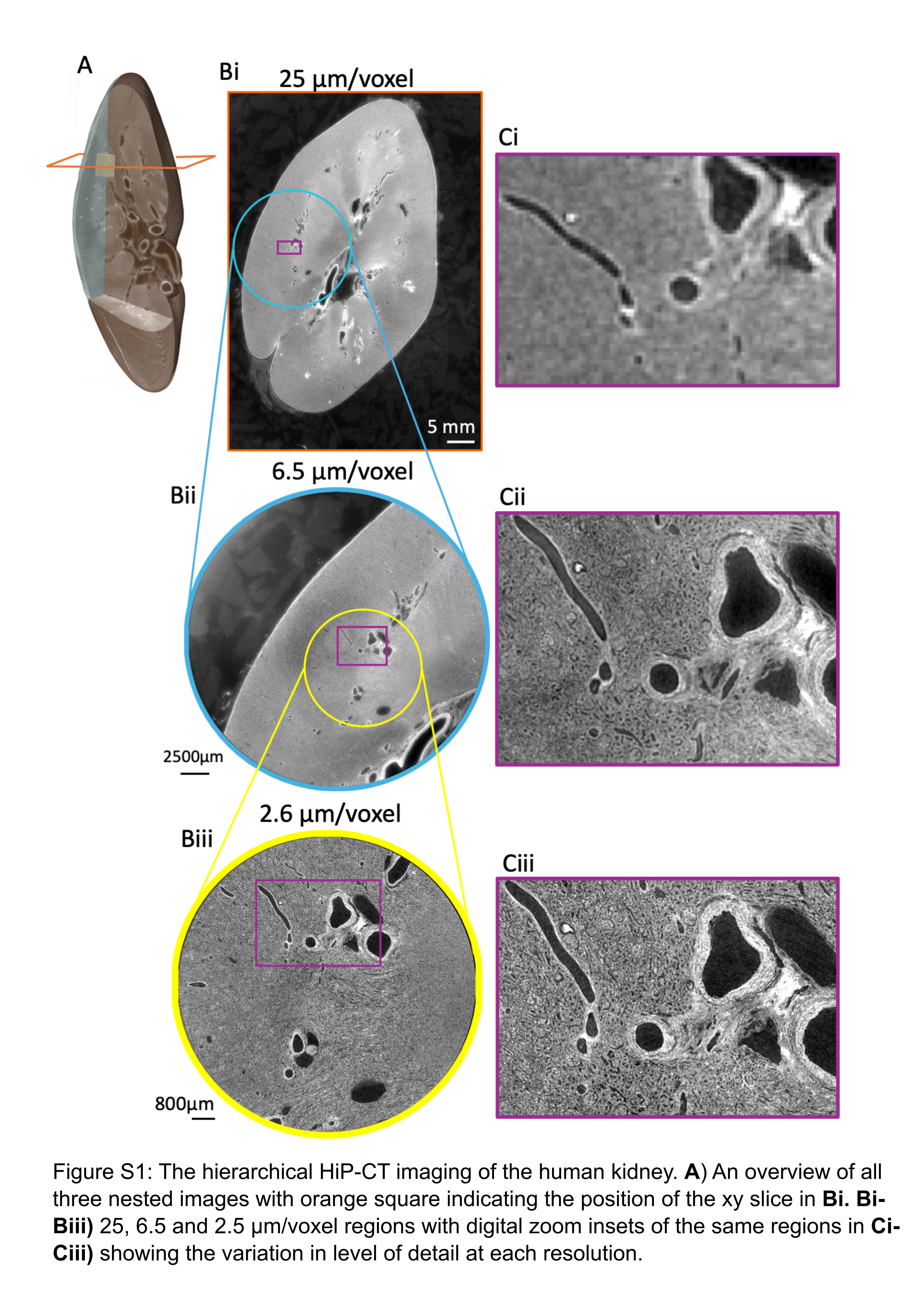

Supplement: Supplement 3 [file media-3.zip › Final_Supplementary_figure_S1.tif]

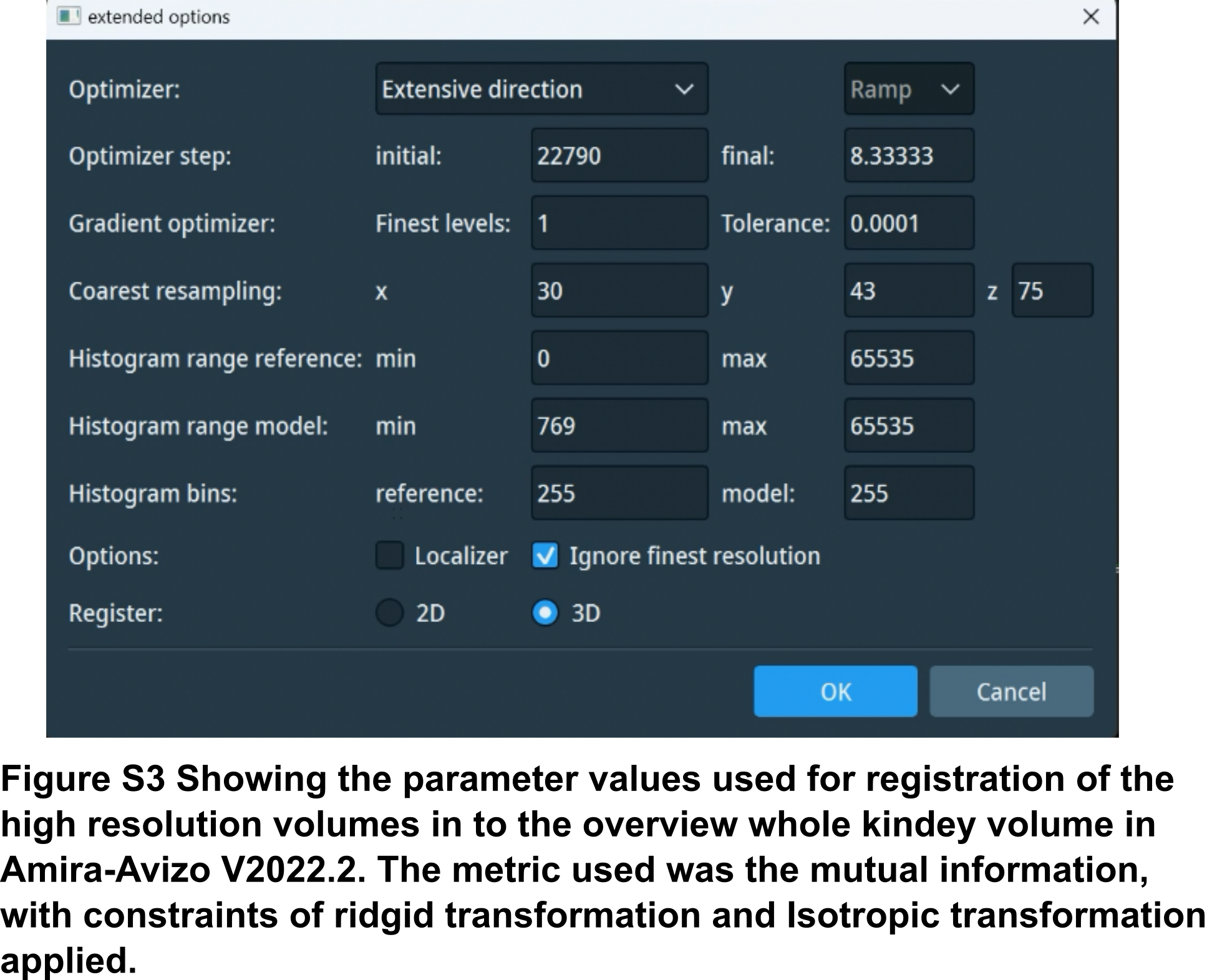

Supplement: Supplement 3 [file media-3.zip › Final_Supplementary_figure_S2.tif]

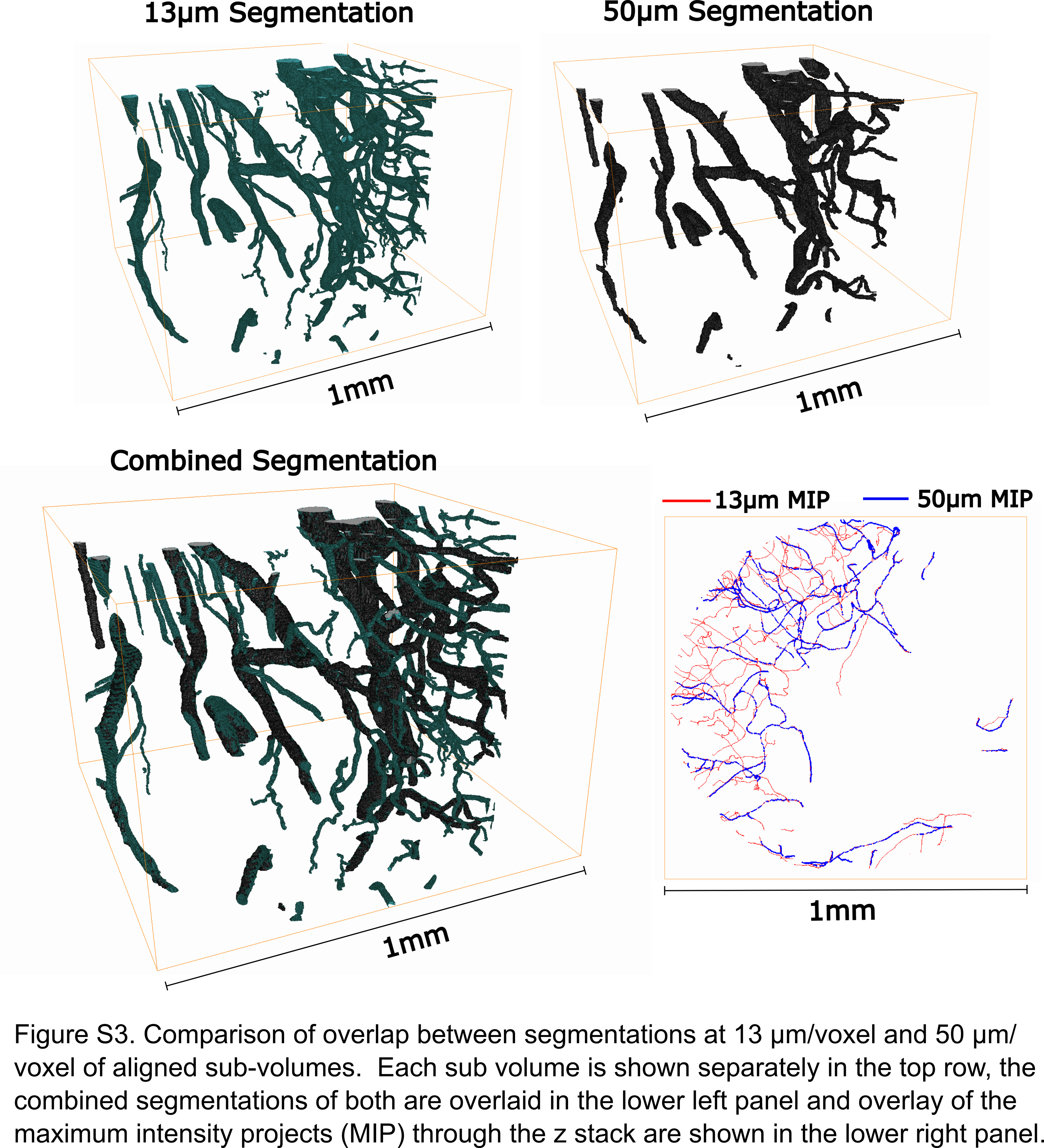

Supplement: Supplement 3 [file media-3.zip › Final_Supplementary_figure_S3.tif]

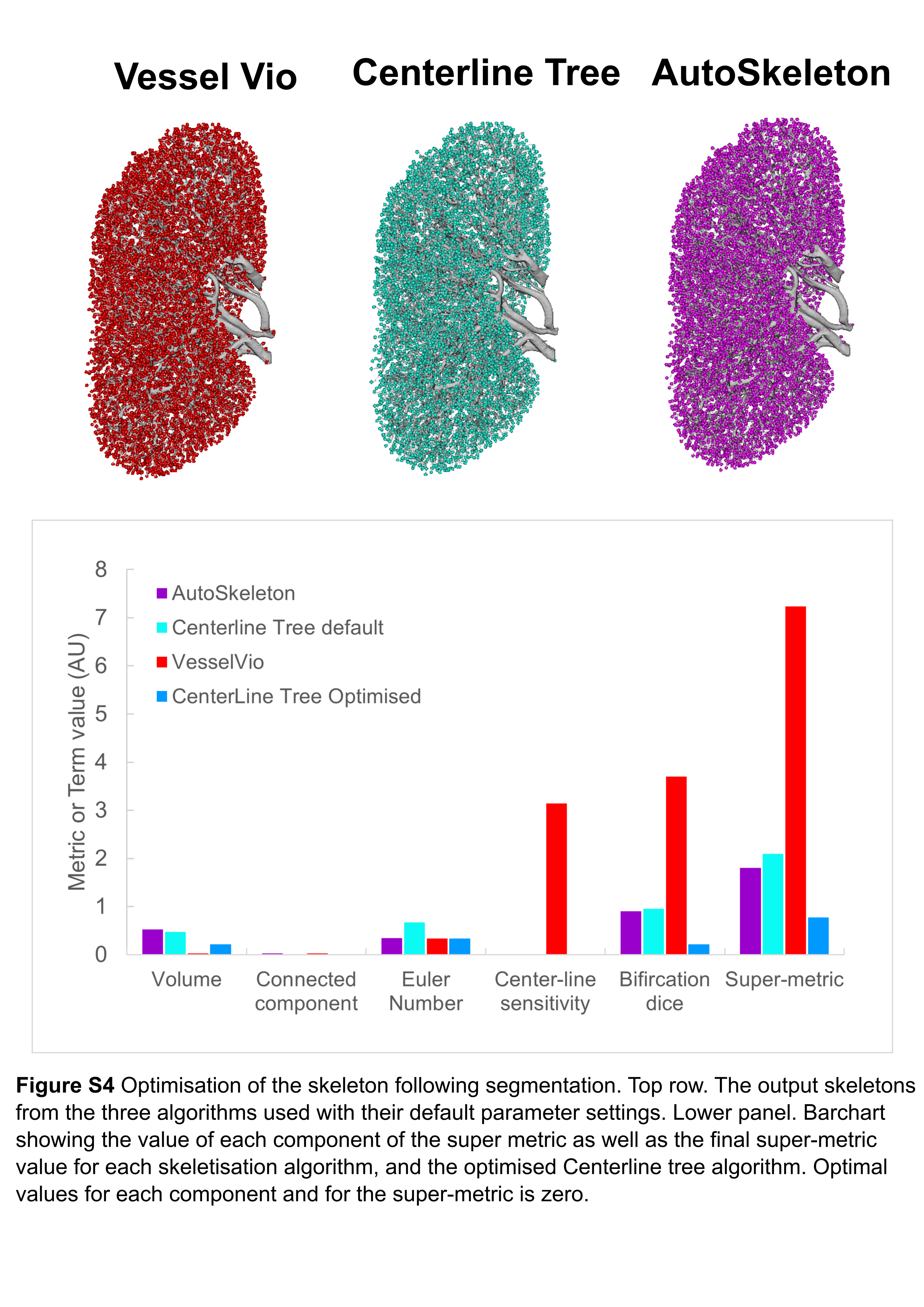

Supplement: Supplement 3 [file media-3.zip › Final_Supplementary_figure_S4.tif]

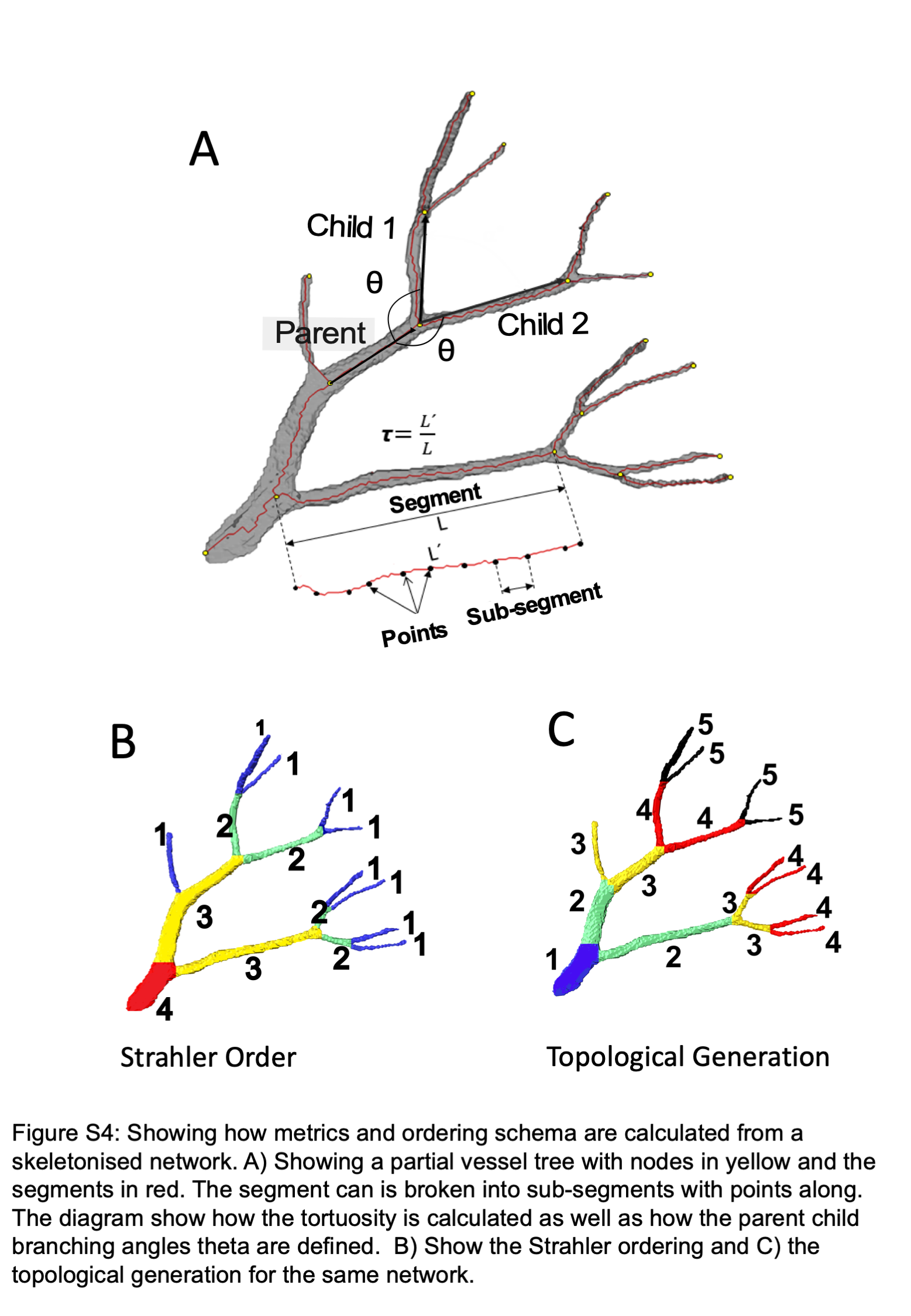

Supplement: Supplement 3 [file media-3.zip › Final_Supplementary_figure_S5.tif]
