# Supplementary material for: Micro to macro scale analysis of the intact human renal arterial tree with Synchrotron Tomography
Source: bioRxiv. 2023 Mar 29:2023.03.28.534566. Preprint. [Version 1] doi: 10.1101/2023.03.28.534566 (PMC10081185; doi:10.1101/2023.03.28.534566)
Supplement: Supplementary file 1 [file media-1.docx]

**Supplementary Information for Micro to macro scale analysis of the intact human renal arterial tree with Synchrotron Tomography**

Rahmani, Shahrokh^1^; Jafree, Daniyal J.^2,3^; Lee, Peter D.^1^; Tafforeau, Paul^4^; Jacob, Joseph^5,6^; Bellier, Alexandre^7^;Ackermann, Maximilian^8,9^; Jonigk, Danny D. ^10,11^; Shipley, Rebecca J.^1^; Long, David A.^2^; Walsh, Claire L.^1^

**Contents**

1. Table S1 ……………. pg1

2. Table S2……………. pg1

3. Figure S1……………. pg2

**Table S1. Metadata for image collection**

| Donor | **PixelSize** | **Beamline** | **Energy (kEv)** | **Propagation Distance (mm)** | **Filters** | **Scintillator** | **Number of projections** | **Exposure Time (s)** | **Sensor name** | **Total scan time** **(hrs)** |
| --- | --- | --- | --- | --- | --- | --- | --- | --- | --- | --- |
| LADAF-2021-17 | 25.00 | BM05 | 81 | 3500 | Mo 0.1 SiO2 8x4mm | LuAG:Ce 1000um | 6000 | 0.035 | PCO edge 4.2 CLHS | 3.7 |

 

 

**Table S2. Hyperparameters of a CNN for segmentation of human kidney zones**

 

| **Hyperparameter** | **Value** |
| --- | --- |
| First layer size | 64 |
| Layers | 5 |
| Patch size | 64 |
| Stride Ratio | 0.5 |
| Batch size | 128 |
| Epochs set | 100 |
| Epochs completed | 63 |
| Loss Function | Categorical Cross Entropy |
| Optimiser | AdaDelta |
| Augmentation | 4 times |
| Augmentation types | Flip horz, Flip vert, rot, shear, scale (90-110%) |
| Learning rate | 1 |
| ρ | 0.95 |
| Epsilon | 1.00E-07 |
| Validation % | 20% |
| Patience | 20 |

**Supplementary Figure 1: (A)** Schematic picture showing blood vessel metrics measured within the human kidney. Vessel: is defined as being between a start and end point; which correspond to either a branching point leading into another vessel branch or a terminal end where no further branches were detectable. Segments: are joining points that discretise the vessel length. Branching angle (α): is defined as the angle between the two vessels, which is measured from the branching point to the ending points of each vessel. Tortuosity (𝞽): is defined as the shortest distance between start and end points of a vessel dividing by the vessel length **(B)** Branching angles distribution (Number of Branching points = 2836, mean = 61.6 ± 29.6°) **(C)** Tortuosity (Number of vessels = 5718, mean = 1.35 ± 0.27) **(D)** Vessel radius against Strahler generation **(E)** Vessel radius over vessel length against Strahler generation
